# γ-Cyclodextrin Metal–Organic Frameworks for Drug Delivery: Current Advances in Synthesis, Activation, Encapsulation and Applications

**DOI:** 10.3390/pharmaceutics18040502

**Published:** 2026-04-18

**Authors:** Lubna Y. Ashri

**Affiliations:** Department of Pharmaceutics, Collage of Pharmacy, King Saud University, Riyadh 11451, Saudi Arabia; lashri@ksu.edu.sa

**Keywords:** biocompatible nanocarriers, γ-CD-MOFs, synthesis, size control, activation, drug encapsulation, drug delivery

## Abstract

Metal–organic frameworks (MOFs) are a versatile class of hybrid crystalline materials that have emerged as promising candidates for a broad range of applications. γ-cyclodextrin MOFs (γ-CD-MOFs) represent an innovative subgroup of MOFs constructed from “edible” γ-CD ligands coordinated with biocompatible metal ions to form an extended porous structure. Owing to their unique characteristics such as their “green” origin, biodegradability, and biocompatibility they became a promising platform for drug delivery applications. Structurally, γ-CD-MOF possess a body-centered cubic structure with dual-mode porosity, enabling the simultaneous encapsulation of hydrophilic and hydrophobic drugs. Such structural features contribute to high loading capacity, tunable release behavior, and enhanced stability of incorporated drugs. In this review, we comprehensively discuss the structural features of γ-CD-MOF, synthesis strategies, crystals size and morphology control, activation and drying techniques, and drug encapsulation approaches. We further address computational and simulation approaches used to predict and optimize drug-framework interactions, as well as post- synthetic modifications aimed at enhancing stability and functionality. The diverse pharmaceutical applications of γ-CD-MOFs are examined, including the delivery of small molecules, macromolecules, multi-drug systems, and emerging pulmonary formulations. Additionally, we examine biocompatibility and safety considerations and current limitations related to aqueous stability, industrial-scale production, and reproducibility. Finally, this review highlights recent progress and underlines future perspectives, emphasizing innovations such as fast drug-loaded MOF formation via spray-drying, co-delivery strategies, and vaccine-oriented formulations. Together, these insights highlight the potential of γ-CD-MOFs to shape the next generation of multifunctional drug delivery systems across interdisciplinary fields.

## 1. Introduction

Metal–organic frameworks (MOFs) belong to a relatively new class of crystalline materials that are constructed by the association of inorganic metal centers with a wide range of polytopic organic ligands [1]. These highly porous hybrid materials possess unique properties that combine the advantages of both organic and inorganic materials such as polymers and mesoporous silica, respectively [2]. Several classes of MOFs have been widely described depending mainly on the choice of the organic linker and the metal ion, each class showing distinct advantages and disadvantages. For example, copper-based MOFs (e.g., HKUST-1) show high surface area but are sensitive to moisture [3], zeolitic imidazolate frameworks (e.g., ZIF-8) exhibit outstanding thermal and chemical stability but relatively limited pore aperture flexibility [4], zirconium-based MOFs (e.g., UiO-66) demonstrate excellent hydrolytic stability though sometimes with lower pore volumes [5], while mesoporous frameworks (e.g., MIL-101) show high loading capacity due to their very large pore sizes but may exhibit stability challenges under certain conditions [6]. Critically, many MOFs are assembled from organic linkers or metal ions that are not biocompatible or may display inherent toxicity [7].

One unique subclass of MOFs is γ-cyclodextrin metal–organic frameworks (γ-CD-MOFs) that is synthesized using γ-CD as the organic linker and a biocompatible metal ion, such as alkali metal cations (mostly K^+^) or, in few instances, transition metals (e.g., Fe^++^) [8,9,10,11,12]. Notably, γ-CD-MOFs address several conventional MOFs limitations by combining the Generally Recognized as Safe (GRAS) status with the intrinsic biocompatibility, biodegradability, and the structural benefits of porous frameworks, which makes them particularly suitable for pharmaceutical applications. To date, the GRAS native CDs (α-, β-, and γ-CDs) are the only carbohydrates known to form MOF crystals [13,14,15]. Structurally, γ-CD-MOFs extended crystalline networks adopt a characteristic body-centered cubic architecture with large pores and channels. In the crystalline lattice, γ-CD internal cavities are hydrophobic, and the interstitial channels and pores are hydrophilic; hence, the framework shows hierarchical dual-mode porosity. These characteristics enable high encapsulation capacity of guest molecules with various sizes and physicochemical properties. Furthermore, compared to traditional MOFs, γ-CD-MOFs uniquely combine the permanent well-ordered porosity of MOFs with the well-known host–guest inclusion complexation ability and biodegradability of CDs [13,14,15]. Due to the unique properties of this “edible” framework, γ-CD-MOFs are positioned at the interface of material science and pharmaceutical technology [8]. The research on γ-CD-MOFs as drug delivery systems is accelerated and their biomedical applications are further reinforced by safety considerations, and intrinsic biocompatibility combined with the relative ease of synthesis [15,16]. Over the past decade, investigations on γ-CD-MOFs have expanded, encompassing multi-drug co-delivery strategies, small drug molecules delivery, macromolecules and biologics encapsulation, and inhalable formulations. Additionally, the pharmaceutical potential of γ-CD-MOFs have broadened due to the advances in post-synthetic modification, crystal engineering, computational modeling, and scalable fabrication methods such as spray-drying.

Regardless of this rapid improvement, various limitations still need to be addressed, including γ-CD-MOFs aqueous instability [17], long-term biosafety [18], industrial scale-up [19] and batch-to-batch reproducibility [20]. Additionally, while general reviews on CD-based MOFs exist [15,18,21], an up-to-date, focused, and critical analytical review granted explicitly to γ-CD-MOFs as drug delivery platforms is presently lacking. Therefore, in this review, a comprehensive and critical analysis of γ-CD-MOFs in drug delivery is provided. Their structural characteristics, synthesis approaches, crystals morphology and size control, activation techniques, drug loading methods, computational insights, and post-synthetic modifications are discussed. Safety considerations and translational challenges are further evaluated, while emphasizing emerging directions such as multi-drug delivery, inhalable systems, and rapid and scalable green fabrication approaches. By integrating recent advances and identifying remaining challenges, this review aims to outline the current state of the field and provides strategic insight to support the rational design of γ-CD-MOFs as next-generation biocompatible drug delivery systems.

## 2. Structure and Physicochemical Characterization of γ-CD-MOFs

**Figure 1:**
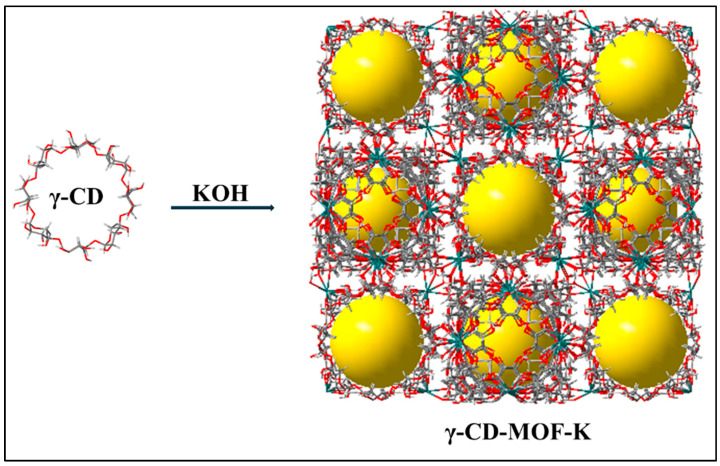
Schematic illustration of γ-CD-MOFs crystal structure and pore system where potassium is the metal ion. K = teal, C = gray, and O = red. Hydrogen atoms and solvent molecules have been omitted for clarity. The largest spheres that can be accommodated in the cavities without touching the van der Waals atoms of the framework are depicted as the yellow spheres [26].

The highly porous three-dimensional (3D) γ-CD-MOF crystals are typically formed by mixing K^+^ with γ-CD ligand where every six γ-CD molecules are connected by metal ions to form a cubic arrangement (Figure 1). These body-centered frameworks feature large central internal cavities with diameters of about 1.7 nm and windows aperture of approximately 0.4–0.8 nm [8]. In addition, the intrinsic cavity of the γ-CD units (~0.78 nm) contributes to a hierarchical dual-porosity system at both the molecular and framework levels [8]. These cages are interconnected through smaller channels at the faces, creating an extended network. The resulting structure possesses the unique advantage of a high specific surface area (SA), with a Brunauer–Emmett–Teller SA (BET SA) of around 719–799 m^2^/g depending on the solvent employed during synthesis [22]. The corresponding solvent-accessible volume has been reported to reach around 80% [22]. These structural parameters have been consistently confirmed by crystallographic and gas adsorption analyses and reported in the literature [22]. Owing to the characteristics of their CD-based organic linker, these materials exhibit hydrophobic internal cavities and hydrophilic exterior surfaces (extended pores of the crystalline structure) resulting from the abundant hydroxyl groups on the CD units. This dual-mode porosity enables the encapsulation of both hydrophilic and hydrophobic drugs [23]. Such structural features enable γ-CD-MOFs to form inclusion complexes with a wide range of guest molecules, and their pores can function as confined nanoreactors [24,25].

Compared to conventional MOFs, γ-CD-MOFs exhibit less topological diversity due to the rigid geometry of CD units. Nonetheless, changes in the coordinating metal ion, salt selection, and synthesis parameters offer structural and functional diversity which dictate framework stability and coordination geometry [27]. The most employed metal ion for constructing γ-CD-MOFs with the classic body-centered cubic topology is K^+^, usually introduced as potassium hydroxide (KOH). Similar to γ-CD, KOH is classified as GRAS by the U.S. FDA under 21 CFR 184.1631 for limited food processing applications. This fact reinforces the suitability of K-γ-CD-MOFs as biocompatible carriers for drug delivery, but further validation through comprehensive physicochemical and biological assessments are still needed to guarantee their intrinsic biocompatibility. Other potassium salts described in the literature and reported to form γ-CD-MOFs include potassium nitrate [28], potassium chloride, potassium bromide, potassium acetate, potassium carbonate, and potassium iodide, all of which lead to the formation of the cubic isostructural frameworks [29,30]. On the other hand, Forgan et al. isolated frameworks with different topologies when Cs^+^ and Sr^2+^ salts were used. They reported isolating two polymorphs assembly under identical synthesis conditions when Cs^+^ salt was used [30]. One polymorph has the typical cubic topology of K-γ-CD-MOFs, while the other polymorph shows a channel structure where γ-CD units are perfectly stacked in one dimension. Additionally, when Sr^2+^ was used, a trigonal “slipped stack” configuration was formed, which resulted in convoluted and nonlinear channel structure [27,30]. Furthermore, a few studies have explored alternative alkali cations by using sodium salts (e.g., sodium hydroxide, chloride and acetate), as well as rubidium salts (e.g., rubidium hydroxide) that yield isostructural frameworks [9,27]. Moreover, in scarce cases ferric nitrate salt was used as a source of iron to synthesize block-like iron based- γ-CD-MOF [9,12,27]. The following table summarizes the key findings discussed above and provides a concise overview of the compared parameters (Table 1).

It is worth noting that γ-CD-MOF crystals are liable to degradation in aqueous media and in physiological environments, which may compromise controlled release of drugs or cause premature leakage [20]. Consequently, considerable efforts have been devoted to enhancing their water stability, thereby enabling certain routes of administration, achieving sustained release, or enabling targeted drug delivery [14]; these strategies will be discussed in Section 8 of this review.

**Figure 2:**
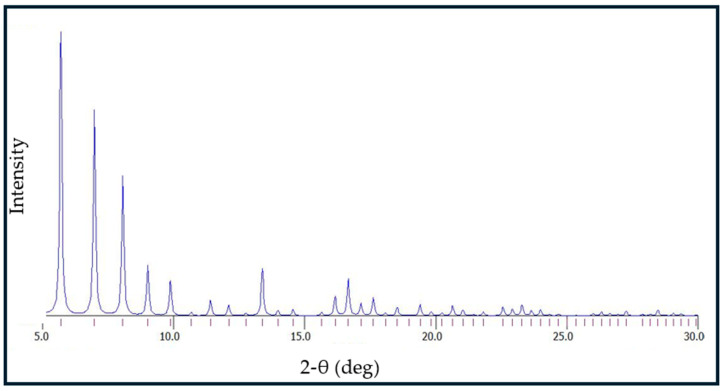
Calculated (simulated) PXRD pattern of γ-CD-MOFs generated from single-crystal X-ray diffraction data [30] acquired using Mercury software (version 3.8, CCDC, Cambridge, UK) [35].

The structural and physicochemical characteristics of γ-CD-MOFs have been studied using several analytical techniques that are widely reported in the literature. One of the most important characterizing methods is the powder X-ray diffraction (PXRD) where characteristic peaks are exhibited by γ-CD-MOFs, which correspond to a body-centered cubic (bcc) structure formed by the coordination of γ-CD units with metal ions [8,33]. Typically, experimental PXRD patterns are compared with calculated or simulated patterns generated from single-crystal X-ray diffraction data (Figure 2) [30] to verify the successful formation, crystallinity and phase purity of the as-synthesized frameworks as well as the stability of drug loaded crystals [26]. Reproducibility of the crystalline structures is also confirmed with the consistent PXRD patterns of γ-CD-MOFs synthesized under similar synthesis conditions. Additionally, changes in position or peak intensity may reflect structural modifications due to amorphization, guest molecule encapsulation, or post-synthetic treatments [34].

To further evaluate structural and physicochemical properties of γ-CD-MOFs, complementary techniques are being implemented besides the PXRD [21]. These include the Fourier-transform infrared spectroscopy (FTIR), which is used to evaluate the functional groups, chemical bonds and the host–guest interactions within the frameworks [33]. Additionally, scanning electron microscopy (SEM) and thermogravimetric analysis (TGA) are also frequently employed to further evaluate morphology and thermal stability of the frameworks, respectively [8,36]. Furthermore, nitrogen adsorption–desorption measurements using a Brunauer, Emmett and Teller (BET) surface area analyzers are used to determine the specific surface area of the γ-CD-MOF [33].

## 3. Synthesis Methods of γ-CD-MOFs

**Figure 3:**
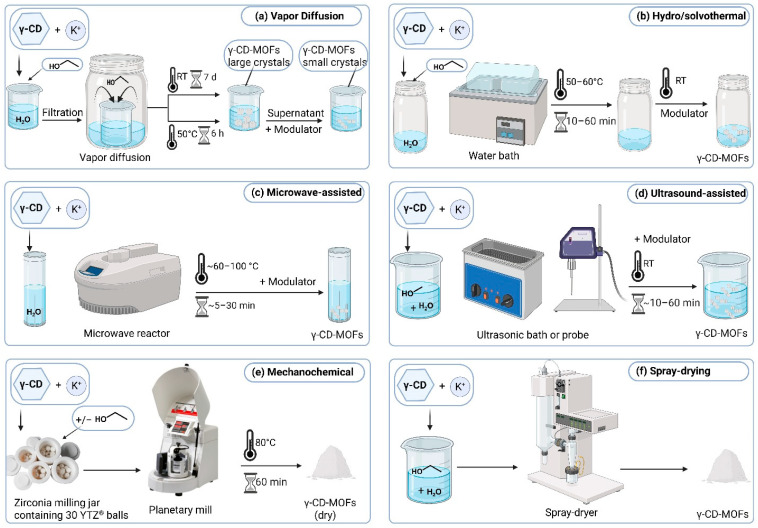
Schematic representations of γ-CD-MOFs synthesis using: (**a**) vapor diffusion; (**b**) hydro/solvothermal; (**c**) microwave-assisted; (**d**) ultrasound-assisted; (**e**) mechanochemical; and (**f**) spray-drying methods. Created in BioRender. Ashri, L. (2026). https://BioRender.com/htzry71, accessed on 17 April 2026.

In CD-MOFs synthesis, crystal size and quality, large-scale production, and batch-to-batch variability remain significant challenges [19,20,33,37]. Therefore, abundant efforts have been made, and several methods have been reported to overcome these challenges and to synthesize monodispersed crystals preferably in the nanorange for drug delivery applications. These methods include, but are not limited to, vapor diffusion, hydro/solvothermal, microwave- and ultrasound-assisted, mechanochemical and spray-drying methods (Figure 3) [38]. Despite differences among these technique, γ-CD-MOF synthesis generally follows the same core steps, which are dissolving γ-CD in water, addition of a highly soluble metal salt, filtration, initiation of nucleation and crystallization, crystal growth (with or without modulators), followed by separation, washing, activation and finally drying the formed crystals.

### 3.1. Vapor Diffusion Method

Vapor diffusion is the first and most commonly used method to prepare γ-CD-MOFs crystals [8,39]. It is simple, mild, safe, avoids harsh conditions, easy to control, and was used initially to prepare micro-sized crystals (200–400 µm) [14,17,40]. It involves dissolving γ-CD and KOH (or other alkali cation) [8,30] at a molar ratio of 1:8 in deionized water, filter the solution through a 0.45 µm filter, to remove any possible impurities or dust, then allow the vapors of a non-polar solvent to slowly diffuse into the solution [41]. Solvent vapors reduce γ-CD-K^+^ complex solubility and enhance the gradual formation and precipitation of high-quality well-defined γ-CD-MOF crystals (Figure 3a). The yield of this method usually ranges between 60 and 85% [42,43].

This method is classically carried out at ambient pressure and temperature where the crystallization process is slow and may take few days up to weeks [8]. Later, modified vapor diffusion method was introduced by Liu et al., where the reaction was carried out at warmer temperatures (50 °C) to shorten the reaction time to just 6 h [20]. This is usually accompanied by the pre-addition of a small amount of the solvent (used for vapor diffusion) into the mother solution before heating [26].

To control the size and achieve homogenous monodispersed γ-CD-MOF crystals, different modulators were utilized such as cetyltrimethylammonium bromide (CTAB), which resulted in generating crystals with diameter range of 1–10 µm [20,44,45]. CTAB addition to the crystallization medium slowed down crystal growth rate, therefore reducing the final crystal size. Lastly, nanoscale γ-CD-MOF (200–300 nm) was achieved when Furukawa et al. added both methanol (MeOH) and CTAB in the crystallization medium [44,46]

Recently, in an effort to rapidly produce γ-CD-MOF crystals and avoid using toxic modulators such as CTAB, “green” seed-mediated method was designed [47]. Here, to control nucleation and growth of the crystals, short-chain starch nanoparticles were used as the seed, which resulted in the formation of a monodispersed crystals that preserved the cubic morphology of γ-CD-MOFs and had the size of about 2 µm. Nevertheless, scaling-down the crystals to nano-range was not possible using this method.

As far as the solvent choice in vapor diffusion method is concerned, it is worth noting that Liu et. al. reported that the choice of the solvent used during γ-CD-MOFs synthesis did not affect the crystal structure but affected its morphological characteristics [20]. They found that using ethanol (EtOH) promoted the formation of hexagon-shaped larger crystals, while MeOH helped form smaller crystals with cubic shape. They attributed this phenomena to the lower boiling point of MeOH that leads to faster vapor diffusion that enables the formation of a large number of nuclei, rapid growth, and the formation of smaller crystals [20]. On the other hand, Oh et. al. found that crystals synthesized using different solvents (MeOH vs EtOH) did not differ greatly and they reported that the most significant difference was the larger size of crystals when EtOH was used [22]. Finally, after collecting the formed crystals, they are washed three times to remove unreacted materials, activated using MeOH, EtOH or isopropanol, then subjected to vacuum drying overnight at around 40–50 °C.

### 3.2. Hydrothermal or Solvothermal Methods

This process is traditionally utilized in MOF crystallization using pressure-resistant sealed vessels (Teflon-lined reactors) at high pressure and elevated temperature above the solvent boiling point [48]. When the solvent used as the reaction media is water, the process is referred to as hydrothermal, while when another organic solvents are used, it is referred to as solvothermal method [49]. Even though this method is a potential route for α-, β-, or γ-CD-MOFs preparation in general, in the literature, no well-documented case was found using the classical hydro/solvothermal method to prepare them. This could be attributed to the risk of decomposition of the large and flexible organic cyclic oligosaccharide of γ-CD that may not stand the harsh conditions of the conventional process. Some studies reported using modified or improved hydro/solvothermal conditions without using elevated temperature or high-pressure autoclave. This was accomplished by preparing γ-CD and KOH stock solution (molar ratio of 1:8) in deionized water and mixing directly with an equal volume of the solvent of choice, then heating in a water bath at around 50 or 60 °C for 10 min up to an hour [50]. A modulator was then added to control the size of the prepared crystals and finally the mixture was left at RT to trigger the deposition of the crystals (Figure 3b) [51,52,53]. The surface morphology characteristic of crystals synthesized by this process showed a uniform cubic crystal with sizes ranging from 3 to 5 µm [49,50]. In several studies, the solvothermal approach has been combined with additional techniques, such as ultrasonication, to synthesize γ-CD-MOFs [54]. For example, Chen et al. dissolved γ-CD and KOH in deionized water, subjected the mixture to ultrasonic treatment at 55 W for 30 min, filtered the solution, and then heated it in a water bath [55]. A modulator was subsequently added before allowing the mixture to stand at RT or in the fridge for crystal formation [14,31,56].

### 3.3. Microwave- and Ultrasound-Assisted Method

In general, and as far as MOFs synthesis is concerned, microwave- and ultrasound-assisted methods have been used to produce nano-sized crystals; however, the monodispersity and homogeneity of the formed crystals were usually difficult to guarantee [57]. Nevertheless, these methods are widely used in γ-CD-MOFs synthesis due to the numerous advantages they present. For example, these methods are rapid, simple, environmentally friendly, inexpensive, energy efficient and produce high yield [49,58]. Therefore, in their study, Liu et al. reported the successful microwave-assisted synthesis of γ-CD-MOFs (Figure 3c), which was employed for drug delivery purposes with the ability to reduce preparation time from hours to minutes [59]. They also reported the ability to tune the size and morphology of the crystals by adjusting the solvent ratio, reaction time and temperature, then they achieved nanometer sized crystals by the aid of polyethylene glycol with a molecular weight of 20,000 g/mole (PEG 20,000) and/or MeOH as modulators [59]. Furthermore, they reported that increasing the reaction time or solvent ratio negatively affected the crystallinity of γ-CD-MOF. It is worth noting that this was the first time PEG 20,000 was used as a modulator to modulate the size and morphology of γ-CD-MOFs crystals [14,17,60]

Similarly, ultrasound-assisted approach has been used for the rapid nucleation and development of γ-CD-MOFs where an ultrasound bath or ultrasonic probe were used (Figure 3d) [17,61]. For example, Wei et al. utilized ultrasonic device quipped with a 6 mm probe that was submerged below the mother solution mixture surface, which was composed of γ-CD and KOH dissolved in water and MeOH. This step was followed by the addition of a modulator and MeOH and the process resulted in the formation of crystals with uniform surface and the typical cubic structure [62]. The uniformity of crystals’ size and morphology can be obtained by optimizing the synthesis parameters including the ultrasound power, reaction time and temperature [63]. For instance, Zhang et al. employed a probe-based ultrasonication at a frequency of 20 kHz and reported the synthesis of 1–2 µm sized crystals through adjusting the synthesis parameters [50]. It is worth noting that the structural integrity of the formed crystals may be adversely affected when using too high ultrasonic frequency (40–50 kHz). Therefore, many reports treated the samples using an ultrasonic frequency of 20–25 kHz [64].

### 3.4. Mechanochemical Method

This method is one of the most simple, economic, and environmentally friendly techniques, which is defined as the chemical synthesis of MOFs that is enabled by applying mechanical force [65]. Here, the main synthesis principle is to grind the solid starting materials with or without minimal amounts of solvent [66]. It is a well-known process in mineral processing and metallurgy but in the last few decades, it is undergoing rediscovery in pharmaceutical field [66]. In 2024, Fujita et al. reported, for the first time, a “wash-free” mechanochemical method to produce highly crystalline γ-CD-MOFs with high SA where the washing step was eliminated [67]. A zirconia milling jar containing 30 YTZ^®^ balls was used and the molar ratio of γ-CD: K^+^: EtOH was equal to 1: 2: 0.04 but can be varied. The mixture was then milled using a planetary mill Pulverisette 6 (Fritsch GmbH, Idar-Oberstein, Germany; supplied by Fritsch Japan, Yokohama, Japan) at a rotation rate of 150 rpm for 5 min (Figure 3e); the products were dried at atmospheric pressure at 80 °C for an hour. Therefore, since the process involved only milling and drying, the final solid yield reached 100%, thereby overcoming scalability constraints and making the process suitable for mass production [67].

### 3.5. Spray-Drying Method

To synthesize MOFs using spray-drying method, the precursor solution is simultaneously injected via the diffuser’s center port with compressed air or nitrogen from the surrounding port (Figure 3f). The droplets are atomized and suspended by the resulting gas flow, which provide heating that leads to rapid solvent evaporation and outward diffusion of the precursor toward the droplet surface. As evaporation continues, the concentration of the precursor increases until reaching supersaturation triggering nucleation, growth, and aggregation of nano-MOF crystals [15]. Tse et al. conducted one of the earliest studies to employ modified spray-drying procedure using an ethanolic precursor for initiating γ-CD-MOFs crystal growth. They demonstrated that the rapid solvent evaporation inherent to the spray-drying process promotes the formation of hollow, spherical MOF particles. These particles exhibit a geometric median diameter (D50) below 5 µm, along with low crystallinity and low density. Such characteristics highlight the potential of spray-dried γ-CD-MOF formulations for use as dry-powder inhalers [68].

Afterward, Kadota et al. showed that varying the properties of the precursor can produce amorphous, partially crystalline, or highly crystalline CD-MOF particles. The studied precursor properties were the EtOH volume ratio, incubation time and precursor concentration [69]. Later in 2025, Tanaka et al. reported the formation of crystalline drug loaded γ-CD-MOF particles using a spray-drying approach [19]. Here, the method proved to be rapid, improved crystals stability, and showed its potential for large-scale production of CD-MOFs for pharmaceutical applications [19].

To conclude, a comparative summary of the above-mentioned synthesis methods, highlighting key differences in reaction conditions, crystal size, advantages, and limitations, is presented in Table 2.

Overall, recent studies present promising progress toward scalable γ-CD-MOF synthesis. Different emerging methods demonstrate the potential for rapid production processes while maintaining structural integrity and crystal homogeneity. Nevertheless, continued investigations are still required to further optimize synthesis conditions, improve reproducibility, and advance translation toward efficient and sustainable large-scale manufacturing.

## 4. γ-CD-MOFs Crystals Size and Morphology Control

The crystal size of γ-CD-MOFs is a crucial parameter that strongly influences their behavior, toxicity, and practical applications [15]. In the last few years, several studies have demonstrated that crystal size can be effectively controlled without compromising crystallinity or porosity by modifying synthesis conditions or using modulators [70]. For example, mixing the γ-CD/KOH mother solution with MeOH in a Teflon autoclave and subjecting the mixture to elevated temperature and pressure (80 °C, 15 h) reduced crystal size from 200 to 400 µm to 10–15 µm [21]. However, γ-CD-MOF crystals are sensitive to harsh conditions such as high temperature, humidity, and polar organic solvents, which may lead to morphological changes and loss of crystallinity [20].

Therefore, Liu et al. used modified vapor diffusion method and reported that the size of γ-CD-MOF crystals can be tuned and regulated by adjusting the reaction parameters including the ratio between precursors, reactants concentrations, temperature, time, type of solvent, and the amount and type of modulators added [20,21]. Among these reaction parameters, the use of modulators or surfactants has proven particularly effective for size control, especially when targeting nanoscale crystals for biomedical applications [14]. CTAB was one of the earliest modulators employed; its addition during vapor diffusion yielded smaller crystals (5–10 µm), while combining CTAB with MeOH further reduced crystal size to the nanoscale (200–300 nm) [25,71,72]. Later, due to the toxicity of CTAB and the risk of polluting the sample, different trials were reported using other modulators such as PEG 6000, PEG 20,000, sodium dodecyl sulfate (SDS), MeOH, EtOH or a mixture thereof [31,40,73]. Depending on the modulator/modulator mix used and the sequence of modulators addition, crystals in the nano-range may be obtained and controlled. For instance, He et al. synthesized nanometer-sized γ-CD-MOF that ranges from 300 to 500 nm and kept their characteristic cubic shape [24]. This was accomplished by stirring MeOH and PEG 20,000 with the γ-CD/KOH mother solution, after subjecting it to vapor diffusion and using a three-blade plastic propeller that rotates at 100 rpm for 20 min. The mixture was then subjected to cold water and kept for 12 h at 15 °C to trigger crystallization. Additionally, Mutlu-Ağardan et al. used vapor diffusion method to prepare γ-CD-MOFs and reported that the sequence of modulators addition may result in crystals with different sizes [45]. Thus, they showed that when PEG 20,000 solution in MeOH was added to γ-CD/KOH mother solution, the size of the produced crystals was 606.3 ± 38.40 nm. On the other hand, when PEG 20,000 powder was first added to the mother solution, followed by the addition of MeOH, the produced crystals were smaller with a size of 337.8 ± 31.86 nm. To date, PEG has emerged as one of the most effective modulators due to its excellent size-control capability combined with low toxicity, making it particularly suitable for biomedical applications [14]. In addition, Qiu et al. reported a seed-mediated crystallization strategy combined with sonication as a rapid and environmentally friendly approach for synthesizing nanoscale γ-CD-MOF crystals. Short-chain starch nanoparticle seeds promoted ordered γ-CD assembly and reduced aggregation, yielding uniform crystals with mean particle diameters of 234–894 nm, depending on sonication time [64]. Furthermore, Zhang et al. used both hydrothermal and ultrasonic-assisted methods to prepare γ-CD-MOF crystals and used different conditions to control the crystals size and morphology [50]. Hydrothermal, ultrasonic, and vapor diffusion techniques are influenced by the same key factors affecting MOF morphology and size, although ultrasound commonly yields smaller particle sizes. These key factors include, but are not limited to, solvent system, metal salt, ligand concentration, and temperature. Overall, effective crystal size control relies on balancing nucleation and crystal growth rates. When crystal growth dominates, larger particles form, whereas suppressing growth, such as through modulator addition, favors nucleation and results in smaller crystals [29]. It is noteworthy that Wang et al. reported the synthesis of both 2D- and 3D-CD-MOF particles, demonstrating that the dimensionality can be tuned by changes in the solvents, type of salt and preparation method [51].

## 5. γ-CD-MOF Crystals Activation and Drying Methods

As mentioned earlier, the formed γ-CD-MOF crystals are collected after synthesis (regardless of the method) and washed few times with a solvent that removes the unreacted materials, modulators, or synthesis solvents. This step is followed by an essential treatment, which is the activation of the frameworks followed by drying. MOF activation is a post-synthetic treatment to remove solvent guest molecules from the internal framework pores while keeping the integrity, porosity, and crystallinity of the MOF [74]. This process helps to render the pores accessible for guest molecules such as drugs, gases or catalytic agents depending on the intended application. Hence, failure to properly activate the framework can lead to a reduced BET SA or cause porous MOFs to appear non-porous [75]. MOF crystals may be activated utilizing different methods including solvent-exchange followed by mild heating or vacuum or supercritical CO_2_ (scCO_2_) activation after which they may be used immediately, stored at 4 °C, or stored over desiccant [39,73,74,76]. The solvent exchange method is the most widely used γ-CD-MOF activation method where it involves using a solvent with a low-boiling point to exchange the occluded solvent in the pores [22]. Different solvents were reported to be used for the activation process including MeOH, EtOH, isopropanol or dichloromethane (DCM). Here, the washed crystals are immersed in the solvent of choice, which is freshly replenished every 24 h for 72 h [39].

On the other hand, scCO_2_ activation is based on using specialized equipment to introduce supercritical CO_2_ fluid to remove occluded solvents without surface tension [73]. Studies have shown that by using this method higher SA and greater pore volume of activated MOFs were observed [20]. Here, the washed MOF precipitates are placed in the reaction chamber, where the temperature is maintained above the critical temperature of CO_2_ (50 °C). Thereafter, scCO_2_ is continuously pumped into the chamber at a pressure of 20 MPa and flow rate of 3 mL/min for 6 h. Finally, the pressure is reduced slowly to atmospheric pressure for 25 min before collecting the activated sample. It is worth noting that supercritical CO_2_ is usually used to activate flexible, hydrogen-bond-rich, or heat-sensitive frameworks [73]; therefore, only few reports used this method in γ-CD-MOF activation.

After γ-CD-MOF activation, proper drying is a critical step because solvent molecules used in framework activation often remain trapped within the pores. Removing these guest species is essential to access the material’s permanent porosity and maximize SA for the intended application. Therefore, after framework activation by solvent exchange, the conventional drying approaches typically involve vacuum drying [22]. Since γ-CD-MOFs are sensitive to heat, applying vacuum lowers the solvents boiling points, therefore allowing their removal at relatively low temperatures, which leads to preserving the framework integrity. This method is gentle, provides controlled removal of the volatile solvent, and reduces the capillary stress within the pores during solvent evaporation, which helps in preventing pores collapse. In addition, vacuum drying limits framework exposure to oxygen and atmospheric moisture, which preserve the crystallinity and reproducibility of the MOF. Recently, advanced drying techniques were used; these include freeze-drying where the frozen solvent is sublimated. This method is gentler, helps maintain the framework integrity, and ensures the complete solvent removal [64]. Furthermore, and as mentioned earlier, scCO_2_ is also used for both activation and drying of the framework [73].

## 6. Drug Encapsulation Strategies

**Figure 4:**
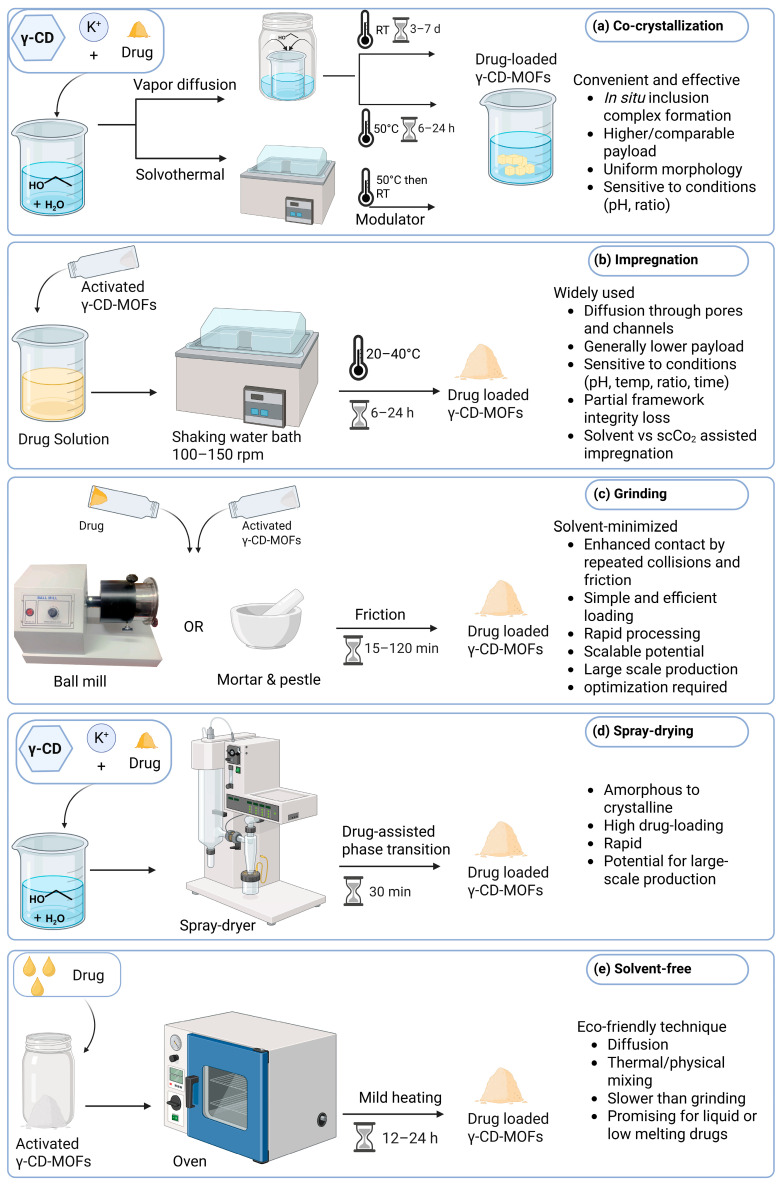
Schematic representations of γ-CD-MOFs drug encapsulation strategies: (**a**) co-crystallization; (**b**) impregnation; (**c**) grinding; (**d**) spray-drying; and (**e**) solvent-free method. Created in BioRender. Ashri, L. (2026). https://BioRender.com/1i4cmz0, accessed on 17 April 2026.

Several methods have been reported for incorporating therapeutic agents into γ-CD-MOFs, with the objective of achieving high drug loading efficiency while preserving the structural integrity of the framework. The selection of loading strategy depends on the physicochemical characteristics of the drug, such as pKa, solubility, and molecular size, as well as the porosity, surface chemistry, and stability of the γ-CD-MOF framework [20]. Additionally, the process parameters (such as the pH, temperature, loading time, and drug/MOF ratio) must be carefully chosen since they have significant effects on the amount of drug loaded [14,26]. Drug encapsulation approaches predominantly involve physical encapsulation through host–guest interactions, including co-crystallization or spray-drying of the pre-synthesized crystals or post-synthetic loading techniques such as solution impregnation, grinding or solvent-free methods (Figure 4).

### 6.1. Co-Crystallization

Drug loading into CD-MOFs via the one pot co-crystallization is a convenient and effective strategy that avoids the use of potentially toxic organic solvents needed sometimes for drug impregnation [14]. Here, the drug is contained into the crystallization medium before the formation of MOF crystals (Figure 4a) [10,14,46,70]. This method has been successfully implemented to load different drugs, including lansoprazole (LPZ), ibuprofen (IBU), and cyclosporin A (CsA), into γ-CD-MOF crystals while maintaining their crystallinity [10,46,71,77]. In many studies, co-crystallization offered higher or comparable payloads when compared to impregnation [14,70]. For example, the loading of LPZ increased from 9.4 wt% via impregnation to 23 wt% using co-crystallization, which can be attributed to the in situ formation of γ-CD–drug inclusion complexes during crystal growth [14]. Additionally, the efficient encapsulation of unstable or poorly water-soluble drugs is enabled using co-crystallization with no structural degradation. The produced crystals have uniform morphology and homogenous drug distribution [14,70]. Nevertheless, and similar to other drug loading strategies, co-crystallization is highly sensitive to drug properties such as molecular structure and crystallization conditions particularly drug-to-γ-CD ratio and the medium pH value [78]. This limits the generalization of this approach across different drug classes and underscoring the need for further systematic investigations [14].

### 6.2. Impregnation

Impregnation, also known as absorption method, is one of the most widely used methods to load drugs into CD-MOFs [25]. It typically involves several steps starting with MOF crystals activation and drying, immersion in drug solution, washing to remove surface-adsorbed drug molecules, and finally drying and collecting the drug loaded crystals (Figure 4b) [10,14]. Using this approach, various drugs such as IBU, LPZ, 5-fluorouracil (5-FU), ascorbic acid, and essential oils have been loaded into CD-MOFs under controlled impregnation conditions [10,23,50,70]. Drug incorporation into CD-MOFs via impregnation has been reported to be highly affected by loading conditions, including the drug/MOF ratio, loading temperature and time, the metal ion constituting the framework and the type of solvent used [9,26,46]. Many systematic studies have assessed solvents effects, naming EtOH, MeOH, acetonitrile, and dichloromethane as the most frequently used media [46]. These studies have shown that solvent polarity strongly governs drug-loading capacity, as demonstrated by IBU and CsA, whose uptake was <5% in nonpolar solvents but increased to approximately 26% in EtOH [14,70]. This phenomenon could be attributed to the favorable host–guest interactions and possible anion-exchange mechanisms involving -OH groups of CD units [14]. Optimization studies based on factorial designs further confirmed that some impregnation conditions significantly affect loading efficiency, as demonstrated for tenoxicam (TNX) in γ-CD-MOFs [26].

It is worth noting that drug loading is commonly quantified either directly or indirectly. The direct method depends on determining drug content within the recovered loaded crystals [26], whereas the indirect method estimates loading from depletion of drug concentration in the supernatant utilizing suitable analytical methods after the separation of drug loaded crystals [9,23]. Despite its simplicity, impregnation generally results in relatively low loading capacities; for example, piroxicam and meloxicam exhibited loadings of only 8.44% and 3.22%, respectively, while paracetamol, metronidazole, and caffeine showed values ≤ 0.3% [14].

Mechanistically, impregnation involves the diffusion of drug molecules through the pre-formed pores and narrow channels of the framework, whereas co-crystallization allows direct assembly of γ-CD–drug complexes into the framework, which generally better preserves structural integrity and crystallinity [14]. In addition, impregnation may lead to the partial impairment of the framework crystallinity due to the progressive degradation in the loading solvent [10,70,78].

In efforts to improve drug loading via impregnation and since the as-synthesized CD-MOFs are typically alkaline, pre-neutralization of the framework before impregnation has been shown to improve the stability of pH-sensitive compounds and enhance the encapsulation efficiency of bioactive molecules [50]. Additionally, scCO_2_-assisted drug loading has been introduced to improve the solubility and diffusion limitations [15]. Compared with conventional solvent-based impregnation, this technique affords substantially higher loading of poorly water-soluble drugs such as honokiol [73].

### 6.3. Grinding

Mechanical grinding, a type of mechanochemical loading, is a simple, efficient, and solvent-minimized method used to encapsulate drugs into γ-CD-MOFs [18,65]. Here, the activated as-synthesized MOF crystals are mixed with drug powder and subjected to grinding (repeated collisions and friction) by a mortar and pestle or ball milling, which lead to intimate contact and enhanced mass transfer of the drug into the porous network (Figure 4c) [14,18]. The encapsulation efficiency is influenced by several factors including drug/CD-MOF molar ratio, grinding duration, temperature, and the use of small amounts of wetting agents to improve molecular contact such as EtOH [14]. In addition, it was reported that temperature often is the most influential variable [18]. This approach has many advantages including its operational simplicity, rapid processing, scalability potential, low solvent consumption and environmental friendliness [18]. It was also reported that this approach enabled relatively high guest incorporation that reached to around 23 wt% for 5-FU and up to 34% for azithromycin [14]. However, performance may vary due to the physicochemical properties of drugs and the control over crystal growth may be limited [18]. Overall, grinding is a practical and efficient drug loading method for γ-CD-MOFs, with potential for reproducibility and large-scale applications. Nevertheless, systematic optimization of grinding conditions is crucial to achieve optimal drug loading and procedural efficiency.

### 6.4. Spray-Drying

In 2025, Tanaka et al. reported using a spray-drying approach to form crystalline drug loaded γ-CD-MOF particles [19]. Here, drug-assisted amorphous–crystal phase transition in γ-CD-MOF was reported where the initially formed amorphous (disordered, non-crystalline) CD-MOFs transformed to crystalline state with the aid of the drugs that were incorporated in the material (Figure 4d). Both model hydrophilic and hydrophobic drugs helped the MOF to reorganize itself into a crystalline well-ordered structure. This can be attributed to the drug molecules promoting nucleation and initiating crystal growth. Additionally, drug molecules may act as molecular linkers and form van der Waals interactions or hydrogen bonds that help in organizing the γ-CD units. Furthermore, drug molecules may stabilize specific packing arrangements that facilitate crystallization, all together contributing to structural formation and enhancing physical stability of the formed MOFs. In general, this method was rapid (≈30 min), improved crystals stability, influenced drug loading that reached >90% *w*/*w*, and showed its potential for large-scale production of CD-MOFs for pharmaceutical applications [19].

### 6.5. Solvent-Free Method

Solvent-free drug loading is an alternative environmentally friendly technique where the use of organic solvents is eliminated. It depends on mixing the drug with the activated as-synthesized MOF crystals and subjecting this physical mixture to mild heating to enable pore penetration (Figure 4e). This technique was successfully applied by Qiao et al. who observed that increasing temperature significantly improved muscone loading into γ-CD-MOFs [56]. This study among others indicate that solvent-free drug loading is promising especially for liquid drugs or drugs with low-melting points, but further studies are required to establish broader generality across different pharmaceutical compounds particularly solids with higher melting points [79].

## 7. Computational and Simulation Approaches for Drug Encapsulation in γ-CD-MOFs

Computational modeling and simulation, including molecular docking, molecular dynamics (MD), and density functional theory (DFT), have become valuable tools for studying and predicting drug encapsulation behavior in CD-MOFs, offering insights that complement experimental studies and help to identify the main physicochemical and structural factors governing drug loading [23,80,81]. Different studies used Monte Carlo-based simulations to model drug adsorption and predict loading capacities in γ-CD-MOFs. For example, simulations of 26 different drug molecules showed that drugs with intermediate molecular weight exhibit higher loading compared to those with large molecular weight due to the steric hindrance [81]. Additionally, it was reported that the presence of benzene rings or halogen atoms enhanced the encapsulation efficiency of the framework. Moreover, it was found that the predicted values closely matched the experimental values of 5-FU loading into γ-CD-MOFs [23,81]. However, deviation between predicted and experimental outcomes may occur since many factors may affect the precision of these computational predictions including the choice of force fields, model assumptions, and simulation conditions [24,33,82]. In many cases, simulations are performed under idealized conditions that do not fully represent pH variations, solvent complexity, competitive interactions with biomolecules, and other physiological conditions. Furthermore, computational analyses have also combined in silico molecular simulations with multiple regression analyses to measure the influence of molecular size, shape, functional groups, and other descriptors on drug–framework interactions [81]. Molecular modeling was earlier applied by Wang et al. to comprehend host–guest interactions in CD systems, highlighting the role of molecular fit and intermolecular forces in complex stability, principles transferable to CD-MOF systems [83]. Fundamental investigations on CD inclusion complexes have likewise utilized computational techniques to clarify non-covalent interactions, thermodynamic profiles, and conformational dynamics between drug molecules and CD hosts, providing valuable insight into their encapsulation mechanisms [80,84]. Together, these computational and simulation strategies accelerate the assessment of encapsulation potential across various drugs and provide a theoretical foundation for rational design of CD-MOF drug delivery systems [15].

Furthermore, to optimize drug loading into γ-CD-MOF systems, statistical design-of-experiments (DoE) approaches such as the Box–Behnken factorial design have also been explored in parallel with molecular modeling. Here, key process variables including drug/framework ratio, impregnation temperature, and time have been systematically evaluated by response surface methodology, which enables the identification of statistically significant parameters affecting drug encapsulation efficiency and provide quantitative assessment of factor interactions [26]. However, although such empirical models can rank the relative importance of impregnation variables and describe general trends, deviations between experimental and predicted outcomes have been reported. This discrepancy could be attributed to the complex host–guest interactions involved in γ-CD-MOF drug loading process, diffusion phenomena and possible structural and crystallinity changes that may not be fully captured by quadratic polynomial models alone [26]. Therefore, integrating molecular-level simulations and statistical optimization tools with experimental validation is critical for advancing predictive reliability and achieving rational design of γ-CD-MOF-based drug delivery systems.

The insights gained from these computational and simulation approaches offer a mechanistic understanding of drug encapsulation and provide a foundation for the practical drug delivery applications in γ-CD-MOFs. Even though the nature of these approaches is mainly predictive, their impact in real-world lies in guiding experimental design, accelerating formulation parameters optimization, and reducing trial-and-error attempts. This directly informs the strategies to enhance their efficiency and stability, which are discussed in the following section, thus showing a clear connection between computational predictions and practical applications.

## 8. Drug Delivery Applications of γ-CD-MOFs and Approaches to Improve Their Stability and Efficiency

Owing to their highly porous crystalline structure that is composed of food-grade γ-CD and alkali metal ions, γ-CD-MOFs are characterized by their biocompatibility, biodegradability, low intrinsic toxicity, tunable host–guest interactions, and amphiphilic cavity structure [10,27,85,86]. Accordingly, they were extensively studied as carriers for a wide spectrum of therapeutics ranging from small drug molecules to biomolecules, volatile compounds, and gaseous therapeutics [45,86,87]. Even though native γ-CD-MOFs have attracted significant attention as drug delivery carriers; recent advances have demonstrated that their modification by surface functionalization, hybrid composite formation, and the incorporation of stabilizing agents may address their inherent limitations [88]. These limitations include their rapid disintegration, aqueous instability, sensitivity to environmental conditions and limited control over release kinetics [15,26]. Modified and functionalized γ-CD-MOFs expanded their intended applications across oral, pulmonary and advanced therapeutic platforms, enabled targeted and stimuli-responsive drug delivery while providing a degree of control over the performance of the framework under physiological conditions [88]. Accordingly, the following sections will demonstrate how both native and modified γ-CD-MOFs perform in different drug delivery settings, which helps in providing a comprehensive understanding of the field.

### 8.1. Performance Improvement and Release Behavior of Encapsulated Drugs in γ-CD-MOFs

A primary function of γ-CD-MOFs in drug delivery is the enhancement of aqueous solubility of hydrophobic drugs, which could be achieved through drug-CD units inclusion complexation combined with the formation of drug nanoclusters inside the framework pores [24]. Additionally, many studies demonstrated the significant improvement of the dissolution rate of these drugs after inclusion into γ-CD-MOFs while their release kinetics can be modified by tuning different factors such as controlling crystal size [26]. Moreover, γ-CD-MOFs enable controlled drug release by reducing burst release and regulating drug diffusion [85]. Furthermore, it was reported that the crystalline lattice of the framework helped in protecting encapsulated drugs from photodegradation, hydrolysis and oxidation. All these properties establish γ-CD-MOFs as effective platforms for improving drug performance and bioavailability.

For instance, γ-CD-MOF substantially improved the apparent aqueous solubility of azilsartan by 340-fold compared to free drug and enhanced its bioavailability [24]. Additionally, Li et al. loaded the anticancer drug triptolide into γ-CD-MOF with high encapsulation [89]. An almost complete release was observed within 12 h with cellular uptake and pharmacokinetic studies demonstrating enhanced bioavailability. Additionally, the anticancer activities of the drug were significantly improved both *in vitro* and *in vivo*. Similarly, Hartlieb et al. have shown that γ-CD-MOFs may form co-crystals with IBU that, when compared to the drug potassium salt, it demonstrated rapid drug absorption with more than two-folds increase in plasma half-life. In addition, the drug hygroscopicity was reduced due to its incorporation into the framework, which offers practical benefits for solid formulations [77]. Likewise, *in vitro* and *in vivo* evaluations in early milestone studies demonstrated reduced toxicity toward normal cells of chemotherapeutics such as 5-FU and carmofur after their encapsulation into γ-CD-MOFs [86]. Other examples of drugs with improved performance due to incorporation into γ-CD-MOFs include methotrexate [43], lansoprazole [41,71], honokiol [73], leflunomide [78], and ketoprofen [90], among others. By studying the chemical structures of several active pharmaceutical ingredients and relating them with their drug payload percentage into γ-CD-MOFs, Liu et al. concluded that compounds containing carboxyl groups displayed higher payload percent. This was attributed to strong electrostatic interactions between the -COO- and K^+^ within the γ-CD-MOFs pores [20]. In contrast, drugs bearing nitrogen-containing or other heterocyclic rings displayed lower adsorption percentages, probably due to steric hindrance that limits effective interaction with γ-CD-MOFs.

Aside from traditional drug payloads, γ-CD-MOFs have been extensively studied to encapsulate natural antioxidants. For example, the encapsulation of curcumin into γ-CD-MOFs protected the compound from degradation caused by UV irradiation with improved dissolution rate and controlled release [52,55]. Furthermore, catechin-loaded γ-CD-MOFs showed improved bioavailability to over 40% in pharmacokinetic studies and improved the compound’s oxidative and photostability [31]. Other examples include the incorporation of caffeic acid and resveratrol into γ-CD-MOFs, which resulted in enhanced stability and controlled dissolution of these compounds, which may improve therapeutic duration and reduce peak-related side effects [63,91].

It is worth noting that the ability of γ-CD-MOFs to encapsulate volatile compounds such as essential oils is one of their unique advantages [92]. These compounds are usually difficult to stabilize in conventional formulations, but studies have shown their ability to be physically retained within the framework and released in a controlled manner. These studies collectively demonstrate that γ-CD-MOFs enable sustained release and are extremely effective in stabilizing labile drugs [93].

As far as drug release from γ-CD-MOF-based systems is concerned, experimental, molecular simulation, and mathematical modeling studies have shown that it is governed by a combination of diffusion-driven, host–guest interaction-mediated, and degradation-assisted methods [94,95]. These processes can be further regulated through system modifications, enabling a more precise control over guest molecules liberation profiles or facilitating stimuli-responsive release behavior [27,70]. The predominant mechanism was found to be diffusion-controlled release through the porous framework, which often shows a biphasic profile that is characterized by an initial burst release of surface-associated drug molecules followed by their sustained release from the internal cavities of the framework [70,94]. This process is strongly influenced by host–guest interactions, crystal size, and surface area [26]. The host–guest interactions within the framework include the inclusion complexation within the CD cavities or the electrostatic attractions between guest molecules functional groups and metal ions and result in slower release kinetics [94]. Furthermore, degradation-controlled release may further lead to drug molecules liberation due to partial or complete framework degradation under physiological conditions [26]. These mechanisms often act synergistically, providing tunable release profiles that depend on post-synthetic modifications and formulation design. Accordingly, various engineering strategies have been developed to modulate these mechanisms, as discussed in the following sections.

### 8.2. Multi-Functional Platforms for Combination and Hybrid Therapies

The amphiphilic nature of γ-CD-MOFs pore structure, comprising both hydrophobic CD cavities and hydrophilic channels, facilitates efficient accommodation of drugs with various physicochemical properties and results in dual-level encapsulation essential for combination therapy strategies. For example, Ohashi et al. showed that γ-CD-MOFs are capable of encapsulating both the hydrophobic 5-FU concurrently with the hydrophilic ascorbic acid enabling their co-delivery [23]. They confirmed the spatially separated loading of the two drugs, experimentally and computationally, which opens new possibilities for synergistic drug action.

Beyond simple co-delivery, another line of exploration combined γ-CD-MOFs with additional functional materials [15]. For example, dissolution studies of sulfasalazine, a drug with low oral bioavailability that is related to its poor water solubility, revealed fast drug release from γ-CD-MOFs under acidic gastric conditions but slower release under neutral conditions. Consequently, Agafonov and colleagues incorporated ethyl cellulose, which effectively diminished the drug burst release, and highlighted strategies to tailor release kinetics [96]. Recent advancements include the enhancement of folic acid apparent solubility by 13,000-fold by incorporating into γ-CD-MOF and SiO_2_ nanocomposites, which provide controlled release features through inorganic reinforcement of the MOF matrix [97]. Additionally, Jia et al. integrated γ-CD-MOF with graphene quantum dots (GQDs) to endow it with strong fluorescence, modified the surface with pH responsive PEG derivatives, and functionalized it with a targeting ligands or aptamer (AS1411) to produce hybrid systems that facilitate targeted doxorubicin (DOX) anticancer therapy. The resulting multifunctional composite highlighted the system suitability for targeted drug delivery by showing high drug loading of around 89%, receptor-mediated targeting, and pH-responsive release [98]. Another example of studying targeted drug delivery utilizing modified γ-CD-MOFs after intravenous administration was reported by He et al. [99]. In this study, cubic γ-CD-MOF nanoparticles were crosslinked and functionalized with the short peptide sequence RGD; then, the low molecular weight heparin and DOX were co-delivered for lung cancer treatment. The system showed an *in vitro* inhibition of migration and invasion of cancer cells and reduced in vivo lung tumor nodule count and spread area, with no signs of normal tissue damage or adverse hematologic effects. These findings strongly support that this novel nanoplatform could be an efficient targeted treatment for lung tumors.

It is worth noting that post-synthetic crosslinking strategies of γ-CD-MOF have been studied to improve the robustness of γ-CD-MOFs while preserving their porous architecture and host–guest encapsulation capability. Among them, the chemical crosslinker diphenyl carbonate [99,100], which is the most widely reported, and ethylene glycol diglycidyl ether were used [32,44]. In addition, surface coating strategies or polymer-assisted crosslinking using polymeric or biofunctional modifiers were also reported including hyaluronic acid [101] poly (acrylic acid) [25], polymers derived from 3,4-ethylenedioxythiophene, and hydrophobic moieties (e.g., C60 or cholesterol) [25].

Generally, drug delivery systems based on CDs have distinctive capacities to form host–guest complexes and assemble supramolecular networks functionalized with tailored ligands, which allow targeted delivery to diseased regions, thereby enhancing therapeutic outcomes while reducing systemic side effects [102]. These systems provide a platform for studying targeted nanocarriers for the efficient stabilization, encapsulation, and delivery of different drug candidates.

An additional promising application of γ-CD-MOFs is in immunomodulation, where they function as immune adjuvants and antigen carriers. Recent studies have shown that modified γ-CD-MOFs (e.g., coated with surfactant) loaded with model antigens such as ovalbumin provoke cytokine secretion and strong antigen-specific IgG responses in vivo [103]. Their crystalline porous structure guards the encapsulated antigens, while the inherent biocompatibility of the MOF provides safe immune cell interactions. Furthermore, the sustained antigen release increases presentation and immune activation compared to fast bolus administration. While less advanced than anticancer delivery, this area highlights the potential of γ-CD-MOFs in adjuvant design and vaccine delivery.

Furthermore, γ-CD-MOFs have been investigated to encapsulate gaseous therapeutics, including NO, H_2_S, and SO_2_ after modifying with surface agents [54]. Controlled release and significant enhancement in thermal stability of these gaseous agents were observed, which mitigate the risks associated with rapid gas release and systemic toxicity. This application highlights the exceptional suitability of γ-CD-MOFs for non-traditional cargos that are poorly compatible with polymeric carriers. In general, these studies demonstrate both the simplicity of “bare” γ-CD-MOF and the potential of multifunctional hybrid systems for drug delivery.

### 8.3. Alternative Routes of Administration Enabled by γ-CD-MOFs: Pulmonary and Inhalation Delivery

Due to their unique porosity, low density, and the hydrophilic outer surfaces that favor interaction with mucosal tissues, recent advances have extended the applications of γ-CD-MOFs beyond oral delivery to alternative administration routes such as transmucosal and pulmonary delivery [52,104]. Drug-loaded γ-CD-MOFs administered through inhalation showed potential for sustained release, controlled aerodynamics and improved local drug concentration in lungs while lowering systemic exposure. Therefore, γ-CD-MOFs were potentially expected to be promising carrier for pulmonary delivery of poorly water-soluble drugs. For example, curcumin exhibited excellent aerodynamic performance after loading into γ-CD-MOFs with improved dissolution rate and elevated wettability [52]. Furthermore, non-invasive drug delivery systems targeting respiratory conditions were introduced where cyclosporine A was loaded into γ-CD-MOF crystals that were optimized with PEG-based modulators [46]. Compared to oral formulations, an increase in bioavailability was reported in repeated inhalation toxicity studies and enhanced *in vitro* aerosol performance was observed. Moreover, broadening of the scope of pulmonary applications was achieved by solidifying D-limonene, a volatile therapeutic agent, within γ-CD-MOFs. Compared to conventional administration routes, this enables efficient dry powder inhalation with controlled particle size, improved stability and systemic bioavailability [105]. These findings, among others, suggest that with proper particle engineering (modulation, size control, and surface coating), γ-CD-MOFs could be promising versatile inhalable carriers for lung-targeted therapy [52].

Collectively, Table 3 summarizes the latest advances in γ-CD-MOF drug delivery according to intrinsic properties or main design strategies instead of individual reports and case studies. This organization stresses the evolution from conventional crystalline porous drug delivery systems to programmable and multifunctional systems, revealing a broader progress in drug delivery applications toward precision-oriented and adaptive platforms. The table integrates γ-CD-MOFs inherent structural characteristics such as host–guest inclusion capability, crystalline nanocavities, and amphiphilic pore architecture with post-synthetic engineering strategies such as surface functionalization, ligand conjugation, crosslinking, multiscale hybridization, and crystal size modulation. It systematically correlates each structural feature or modification strategy with its mechanistic basis underlying enhancements in stability, solubility, release control, and targeting, while drawing the translational path of γ-CD-MOFs toward nanotherapeutics that are clinically relevant.

The above-mentioned structural engineering strategies directly affect γ-CD-MOF systems pharmaceutical performance and biological behavior. Therefore, Table 4 summarizes the major therapeutic applications facilitated by these modifications.

## 9. Biocompatibility, Safety, and Strategies to Overcome Current Challenges in γ-CD-MOF-Based Drug Delivery

Unlike many traditional MOFs that incorporate potentially toxic metal centers or synthetic organic linkers, the “green” nature of γ-CD-based MOFs and their degradation into non-toxic CD monomers and physiologically innocuous metal ions suggest a high safety profile and biocompatibility [106]. This inherent degradability not only reduces long-term toxicity concerns and facilitates broader biomedical application but also aligns with regulatory expectations for biodegradable nanocarriers [36]. Different investigations have shown satisfactory behavior of γ-CD-MOFs in both *in vitro* and *in vivo* studies, which is generally vital for drug carriers [9]. Multiple *in vitro* cytotoxicity assays have addressed these aspects for γ-CD-MOF and showed that, even at elevated concentrations, they exhibit low intrinsic toxicity toward different mammalian cell lines with insignificant adverse effects on function and morphology [36,107]. For example, when utilized as carriers for gasotransmitters (e.g., NO), γ-CD-MOFs demonstrated low cytotoxicity, with more than 95% cell survival rates even at high concentrations (~1.2 mg/mL) [54]. In addition, loading drugs into γ-CD-MOFs was demonstrated to reduce their toxicity toward normal cells. For example, in a study encapsulating 5-FU, carmofur and salicylic acid, a reduction the *in vitro* cytotoxicity, hepatotoxicity, and neurotoxicity was observed on fibroblasts, liver cells and neural cells, respectively [86]. Moreover, developmental toxicity and abnormalities in an in vivo model (zebrafish) was lower compared with the free drug [86]. Such protective effects confirm both the biocompatibility of the framework and its capacity to alleviate drug-induced toxicity in biological systems [86]. Furthermore, in vivo pulmonary delivery studies using repeated administration of drug-loaded γ-CD-MOFs have reinforced their safety profile, showing only minimal inflammatory responses with no meaningful increase in pro-inflammatory markers such as IL-4 and TNF-α [46]. In addition, there was no significant histological damage or alteration in major organs.

Collectively, the available evidence from different studies supports γ-CD-MOFs as highly biocompatible nanocarriers with low toxicity and strong potential for drug delivery; however, converting these outcomes into clinically safe systems necessitates addressing a number of key challenges. It is worth noting that these findings are mainly derived from studies that are limited to *in vitro* assessments or preclinical in vivo models, and, up to date, no γ-CD-MOF-based formulations have proceeded to clinical trials or received regulatory approval. While *in vitro* and in vivo studies generally report minimal γ-CD-MOFs toxicity, upcoming investigations must define safe therapeutic dose windows in human tissues and study whether potential interactions between γ-CD-MOFs and drug payloads could produce unpredicted side effects in the biological milieu [15,46]. These assessments are mostly important because the in vivo environment may alter drug release behavior, the framework stability, and immune recognition in ways not fully captured by current models, highlighting the need for more systematic and regulatory-oriented safety studies [86].

**Figure 5:**
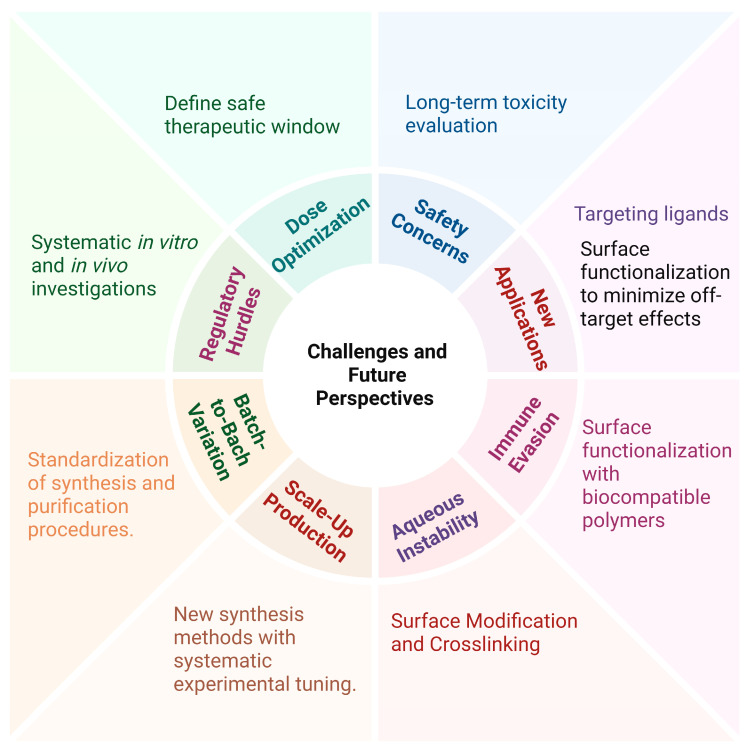
Challenges and future perspectives of γ-CD-MOFs in drug delivery applications. Created in BioRender. Ashri, L. (2026). https://BioRender.com/z5zpx22, accessed on 17 April 2026.

To facilitate clinical translation and overcome the remaining safety challenges, as well as limitations related to aqueous instability, scalability, and batch-to-batch variability, several strategies may be effective and could be considered. For example, defining safe therapeutic windows by prioritizing systematic investigation of dose–response relationships and long-term toxicity in relevant animal models [82]. Additionally, reducing premature degradation and improving structural stability under physiological conditions, particularly in aqueous environments, by the rational design of γ-CD-MOFs through crosslinking and surface modification [86,108]. Furthermore, minimizing immune recognition and off-target interactions by surface functionalization with biocompatible polymers or targeting ligands [82,98,99]. Finally, addressing scalability challenges and minimizing batch-to-batch variability by standardization of synthesis and purification procedures, which is critical to ensure reproducibility and facilitate regulatory approval. Collectively, these approaches can support the safe-by-design development of γ-CD-MOF-based drug delivery systems and accelerate their progression toward clinical applications (Figure 5).

## 10. Conclusions and Future Directions

Collectively, γ-CD-MOFs have shown significant potential as versatile nanocarriers drug delivery platform due to their unique structural and physicochemical properties. Their structure integrates the hydrophobic γ-CD cavities with the hydrophilic framework pores, which enable multi-drug or dual-mode of encapsulation of hydrophilic and hydrophobic agents. Experimental studies have shown that γ-CD-MOFs can incorporate a wide range of therapeutic entities ranging from small molecules and natural antioxidants to complex biologics and gasotransmitters, highlighting their potential in next-generation delivery systems. Compared to native γ-CD, γ-CD-MOFs show superior drug loading capacity and more controlled release properties under physiological conditions, along with improved solubility, bioavailability, and stability of incorporated drugs. Latest applications of γ-CD-MOFs have extended from the conventional oral and parenteral systems to include advanced inhalable formulations for pulmonary delivery, where they enhanced the bioavailability of loaded drugs and showed acceptable safety profiles upon repeated-dosing. Furthermore, *in vitro* and *in vivo* assessments in cell lines and model organisms have supported the low toxicity and favorable biocompatibility of γ-CD-MOFs, highlighting their protective and safe interaction with biological systems relative to free drugs.

It is worth noting that despite these advances, several challenges remain. One of the most important challenges is the aqueous instability of γ-CD-MOFs in physiological environments, as the coordination bonds may undergo rapid dissolution, resulting in premature drug release and loss of structural crystallinity and integrity. Strategies have been explored to enhance stability including post-synthetic surface modification and incorporation of biocompatible additives; however, further optimization is still needed. Ongoing innovations—including hybrid composite systems, immunomodulation, inhalable formulations, and targeted delivery strategies—reflect the development of γ-CD-MOFs from conceptual “green MOFs” into a multifunctional and highly adaptable drug delivery platform.

Nevertheless, critical aspects such as comprehensive systemic safety in human tissues, long-term toxicity data, elucidation of biodegradation pathways, batch-to-batch reproducibility, and balancing high loading capacity with controlled release remain insufficiently addressed and warrant further investigations. Finally, the transformation of γ-CD-MOFs from laboratory investigations to clinical applications and large-scale industrial production will benefit from guided synthesis procedures, pragmatic encapsulation schemes suited to specific drug properties, and the integration of innovative computational tools (such as machine learning-assisted design) to predict and tune performance reliably. Eventually, sustained interdisciplinary efforts that combine material science, pharmacy, and regulatory considerations will be crucial to fully realize therapeutic potential of γ-CD-MOFs as next-generation drug delivery systems.

## Figures and Tables

**Table 1:** Metal ion, directed structural evolution and pharmaceutical relevance of γ-CD-MOFs.

Metal Ion (Source)	Topology	Relevant Property; Application	References
K^+^ (KOH, KNO_3_, KCl, KBr, KI, KOAc, K_2_CO_3_)	Classical cubic body-centered	Highly porous 3D framework with large spherical central pores that are interconnected with smaller channels; widely used in drug delivery	[9,11,28,29,30,31,32]
Cs^+^ (CsOH)	Two polymorphs: (I) Cubic (isostructural to K-based system) (II) 1D channel polymorph	(I) Structural variant with potentially altered pore characteristics (II) γ-CD units perfectly stacked in one dimension to form 1D channel; limited biomedical exploration	[11,30]
Sr^2+^ (SrBr2)	Trigonal “slipped stack” configuration	Convoluted, nonlinear channels due to divalent coordination	[27,30]
Na^+^ (NaOH, NaCl, NaOAc)	Cubic (isostructural to K-based system)	Lower stability than K-based system with a slightly different pore volume	[9,30,32]
Rb^+^ (RbOH)	Cubic (isostructural to K-based system)	Potential differences in pore size/thermal stability; limited application data	[11,30]
Fe^3+^ (Fe(NO_3_)_3_)	Block-like framework with distinct coordination chemistry; non-classical topology	A non-alkali node in a γ-CD framework. Higher porosity and enhanced drug loading in some cases, but not standard for drug delivery; Functional potential (magnetic/catalytic)	[9,12,27,32]

**Table 2:** Comparative summary of synthesis methods for γ-CD-MOFs: conditions, advantages, and limitations.

Method	Conditions	Time	Crystal Size	Advantages	Limitations	Refs.
Vapor diffusion	RT, mild heat	Hours, days or weeks	200–400 µm to nm with modification	Simple, mild, safe, easy to control, high crystallinity	Slow, not scalable	[8,17,20,40,44,46]
Hydro/ solvothermal	Moderate heat	Minutes to hours	3–5 µm	Faster than vapor diffusion	Limited reports, possible degradation	[14,31,50,51,52,53,56]
Microwave- assisted	Elevated temperature	Minutes	nm to µm	Rapid, simple, energy efficient, environmentally friendly, inexpensive, tunable, high yield	Reproducibility issues	[14,17,49,57,58,59,60]
Ultrasound- assisted	RT, moderate heat		1–2 µm		Risk of structural damage with high frequencies	[17,50,61,62,63]
Mechanochemical	Solid-state	Minutes	Variable sizes	Green, scalable, 100% yield, suitable for mass production	New, limited studies	[65,66,67]
Spray-drying	High temperature gas	Seconds	Less than 5 µm	Rapid, scalable, industrial potential	Crystallinity variability depending on reaction conditions	[19,68,69]

**Table 3:** Major intrinsic properties and engineering strategies shaping γ-CD-MOF-based drug delivery platforms.

Intrinsic Property/Engineered Strategy	Mechanistic Basis	Therapeutic Advantages Structural/Physicochemical Outcome	Examples [References]
Host–guest inclusion complexation and nanocluster formation within crystalline cavities	Supramolecular encapsulation via non-covalent interactions	Enhance solubility, modify diffusion kinetics and dissolution rate, reduce burst release, improve bioavailability, and protect labile drugs from photodegradation, hydrolysis, and oxidation	Azilsartan [24]
			Lansoprazole [41,71]
			Methotrexate [43]
			Honokiol [73]
			Ibuprofen [77]
			Leflunomide [78]
			Triptolide [89]
Amphiphilic pore architecture	The simultaneous encapsulation of hydrophobic and hydrophilic agents within the crystalline framework	Enable synergistic therapy and dual cargo loading	5-flourouracil and ascorbic acid [23]
Surface functionalization	Alters surface chemistry and charge via ligands or polymers	Enable prolonged circulation, modify cellular interaction, biodistribution, protein adsorption, colloidal stability, and stimuli responsiveness	PEGylated or peptide-decorated γ-CD-MOFs systems [98]
Targeting ligands conjugation	Selective binding enabled by receptor-specific ligand attachment	Enable biological specificity, targeting capability and receptor-mediated internalization	Aptamers- [98] or RGD-functionalized [99] γ-CD-MOFs systems
Crosslinking	Reinforce the framework by introducing covalent/coordination bonds or polymer-mediated networks	Improve structural robustness and aqueous stability, regulate framework degradation and drug release	Ethylene glycol diglycidyl ether [32,44]
			Diphenyl carbonate [99,100]
			Biofunctional modifiers (e.g., hyaluronic acid) [101]
			Poly (acrylic acid), 3,4-ethylenedioxythiophene, and hydrophobic moieties (C60, cholesterol) [25]
Hybridization with polymeric matrices and inorganic nanoparticles	Composite systems provide synergistic physicochemical interactions and facilitate dynamic structural tuning	Improve biocompatibility, enhance mechanical strength, and tunable release kinetics. Provide fluorescence, enable imaging or stimulus responsiveness	Sulfasalazine with ethyl cellulose [96]
			Folic acid incorporating into γ-CD-MOF and SiO_2_ nanocomposites [97]
			Integrated γ-CD-MOF with graphene quantum dots for doxorubicin delivery [98]
Crystal size adjustment	Crystals’ dimensions are modulated by controlling nucleation and growth	Impact surface-area-to-volume ratio and diffusion pathways, influence drug loading capacity, and release kinetics	Micrometer and nanometer sized crystals [26]

**Table 4:** Application-specific therapeutic outcomes of engineered γ-CD-MOF: from mechanistic basis to clinical function.

Therapeutic Application	Application-Specific Mechanistic Basis	Therapeutic Advantages	Examples [References]
Immunomodulation, biological and vaccine delivery	Protect antigens within the crystalline matrix, enable sustained release, stimulate antigen-specific IgG responses and cytokine secretion in vivo.	Reduce toxicity, enhance immune response, improve antigen presentation with vaccine adjuvant potential	Antigen-loaded γ-CD-MOF vaccine systems [86,103]
Pulmonary, non-oral, and non-invasive delivery	Particle engineering approaches (size modulation, PEGylation, surface coating) lead to low density, surface wettability, improve aerosol performance and dissolution behavior	Facilitate interaction with mucosal and pulmonary membranes, improve local bioavailability, and lung-targeted therapy	Inhalable γ-CD-MOF therapeutics and transmucosal nanomedicine delivery systems of curcumin [52]
			cyclosporine A [46]
			D-limonene [105]
Stabilization of volatile and non-traditional therapeutics after surface modification	Physical sequestration of chemically unstable gaseous, volatile, or biologically sensitive molecules within the crystalline lattices	Enable controlled gas release, improve thermal stability, reduce systemic toxicity associated with rapid release. Facilitate pharmaceutical application of therapeutic classes previously limited by stability or delivery constraints	Gas therapy (NO, H_2_S) [54], natural bioactives, and essential oil stabilization systems and delivery platforms [92]
Targeted cancer therapy	Receptor-mediated uptake and tumor microenvironment-responsive release	Reduced off-target toxicity, enhanced intracellular delivery	Ligand-functionalized γ-CD-MOF systems Triptolide [89]
			5-flurouracil and carmofur [86]
			Doxorubicin [98]

## Data Availability

No new data were created or analyzed in this study. Data sharing is not applicable to this article.
